# Classification of autism spectrum disorder using electroencephalography in Chinese children: a cross-sectional retrospective study

**DOI:** 10.3389/fnins.2024.1330556

**Published:** 2024-01-25

**Authors:** Si Yang Ke, Huiwen Wu, Haoqi Sun, Aiqin Zhou, Jianhua Liu, Xiaoyun Zheng, Kevin Liu, M. Brandon Westover, Haiqing Xu, Xue-jun Kong

**Affiliations:** ^1^Anthinoula A. Martinos Center, Massachusetts General Hospital, Charlestown, MA, United States; ^2^Hubei Maternity and Child Health Hospital, Wuhan, Hubei, China; ^3^Department of Neurology, Beth Israel Deaconess Medical Center, Boston, MA, United States; ^4^Huangshi Maternity and Child Health Care Hospital, Huangshi, Hubei, China; ^5^Department of Biomedical Informatics, Harvard Medical School, Boston, MA, United States; ^6^Department of Neurology, Massachusetts General Hospital, Boston, MA, United States; ^7^Department of Psychiatry, Beth Israel Deaconess Medical Center, Beth Israel Deaconess Medical Center, Boston, MA, United States

**Keywords:** autism spectrum disorder, electroencephalography, machine learning, spectral power, functional connectivity, coherence

## Abstract

Autism spectrum disorder (ASD) is a complex neurodevelopmental condition characterized by diverse clinical features. EEG biomarkers such as spectral power and functional connectivity have emerged as potential tools for enhancing early diagnosis and understanding of the neural processes underlying ASD. However, existing studies yield conflicting results, necessitating a comprehensive, data-driven analysis. We conducted a retrospective cross-sectional study involving 246 children with ASD and 42 control children. EEG was collected, and diverse EEG features, including spectral power and spectral coherence were extracted. Statistical inference methods, coupled with machine learning models, were employed to identify differences in EEG features between ASD and control groups and develop classification models for diagnostic purposes. Our analysis revealed statistically significant differences in spectral coherence, particularly in gamma and beta frequency bands, indicating elevated long range functional connectivity between frontal and parietal regions in the ASD group. Machine learning models achieved modest classification performance of ROC-AUC at 0.65. While machine learning approaches offer some discriminative power classifying individuals with ASD from controls, they also indicate the need for further refinement.

## 1 Introduction

Autism spectrum disorder (ASD) is characterized by a range of neurodevelopmental disabilities affecting an individual’s social interactions, communication, behavior, learning, and overall functioning [National Institute of Mental Health (NIMH), 2022]. The diagnosis of ASD currently relies primarily on developmental and behavior screenings, as there are no definitive medical tests available [Centers for Disease Control and Prevention (CDC), 2022]. Researchers have recently turned their attention to the potential of utilizing neuroimaging modalities, such as electroencephalography (EEG) biomarkers to enhance ASD diagnosis. These EEG biomarkers hold promise as objective tools for early screening and intervention, shedding light on the underlying neural processes associated with ASD (Gurau et al., 2017). Several studies have explored EEG characteristics, such as spectral power and functional connectivity across various frequency bands, in children with ASD (Brihadiswaran et al., 2019; Das et al., 2023). Although certain studies consistently demonstrate notable distinctions in spectral power and functional connectivity between autistic and typically developing children, conflicting results have also been reported (Garcés et al., 2022). These inconsistencies can be seen even in the well supported U-shaped profile of electrophysiological power changes noted in children with ASD as compared to controls (Wang et al., 2013). As described by Wang and colleagues, the U-shaped profile of EEG power encompasses increased spectral power at high frequency (beta, gamma) and low frequency (delta, theta) bands, with reduced power noted in the alpha band. However, several studies have revealed inconsistencies in this U-shape pattern of EEG spectral power, with reduced power in ASD individuals and enhanced (Zhao et al., 2023) or unaffected alpha power (Liao et al., 2022) in most brain regions. In addition, EEG coherence studies with ASD individuals demonstrate similar inconsistencies across various frequency bands, with alterations in local and long-distance coherence within and between different brain regions and hemispheres (Milovanovic and Grujicic, 2021). Thus, overall, no consensus has been achieved within the ASD research community. This underscores the importance of conducting a data-driven analysis on a sizable sample of young children with ASD aiming to identify the EEG functional connectivity/coherence specific features and their relationship with ASD core symptoms, the neurobiology basis behind, and the potential value for ASD early diagnosis and subtyping.

In this study, we analyzed resting-state EEG from a total of 288 children, comprising 246 with ASD and 42 controls. Our approach extracted diverse EEG features related to spectral power, functional connectivity, complexity, and time domain statistics. Our primary objective was to identify differences in these EEG features between the ASD and control groups, employing statistical inference and machine learning methods.

## 2 Methods

### 2.1 Cohort and study design

The study was a retrospective cross-sectional study conducted over a period of 3 years and 1 month, from October 2013 to November 2016, at the Hubei Provincial Women and Children’s Hospital in Wuhan, China. The study initially included a total of 362 participants with clinically diagnosed ASD and 57 health control participants. ASD participants were recruited from children that were visiting the neurology department for ASD and other neurological disorders. On the other hand, control participants were recruited from children that came to the hospital for routine physicals (i.e., well-child visits). ASD diagnoses were made from a team of two clinicians based upon The Diagnostic and Statistical Manual of Mental Disorders, Fifth Edition (APA, 2013) and Childhood Autism Rating Scale second edition (Schopler, 2010). Participants with neurological organic lesions, genetic metabolic diseases, hearing impairment, and psychiatric disorders were excluded from the study. 121 participants who had their eyes closed were excluded from the study due to the heterogenous electrophysiology between individuals with eyes open and those with eyes closed. Moreover, the substantially higher number of participants with eyes open, in contrast to those with eyes closed, contributed to this decision. Table 1 provides information on the final cohort of 288 participants included in the analysis.

**Table 1:** Participant characteristics.

	ASD (*N* = 246)	NO ASD (*N* = 42)	Overall (*N* = 288)
**Sex**			
Female	32 (13.0%)	12 (28.6%)	44 (15.3%)
Male	214 (87.0%)	30 (71.4%)	244 (84.7%)
**Age months**			
Mean (SD)	36.4 (14.0)	38.7 (13.9)	36.8 (13.9)
Median [Min, Max]	34.0 [12.0,150]	36.0 [22.0,81.0]	34.0 [12.0,150]
**Height (cm)**			
Mean (SD)	97.4 (8.25)	99.8 (8.21)	97.8 (8.27)
Median [Min, Max]	96.4 [80.3, 124]	97.9 [80.0, 117]	96.7 [80.0, 124]
Missing	86 (35.0%)	9 (21.4%)	95 (33.0%)
**Weight (cm)**			
Mean (SD)	15.5 (4.21)	15.7 (1.95)	15.5 (3.91)
Median [Min, Max]	14.7 [10.0, 48.9]	16.1 [10.1, 19.5]	15.0 [10.0, 48.9]
Missing	86 (35.0%)	9 (21.4%)	95 (33.0%)
**BMI (kg/m**^**2**^**)**			
Mean (SD)	16.2 (2.97)	15.8 (1.48)	16.1 (2.78)
Median [Min, Max]	15.9 [12.9, 47.0]	15.9 [13.0, 18.9]	15.9 [12.9, 47.0]
Missing	86 (35.0%)	9 (21.4%)	95 (33.0%)
**Childhood Autism Rating Scale**			
Mean (SD)	32.8 (6.12)	25.1(4.09)	31.7 (6.46)
Median [Min, Max]	32.0 [12.0, 97.5]	26.0 [13.0, 29.0]	30.0 [12.0, 97.5]
Missing	15 (6.1%)	3 (7.1%)	18 (6.3%)
**Intelligence Quotient (IQ)**			
Mean (SD)	61.2 (8.20)	65.1 (10.8)	61.8 (8.79)
Median [Min, Max]	57.0 [50.0,83.0]	62.5 [50.0, 86.0]	57.0 [50.0, 86.0]
Missing	101 (41.1%)	12 (28.6%)	113 (39.2%)

### 2.2 Institutional review board approvals

All participants included in the study were given informed consent by their parents and guardians subject to oversight and approval by the ethics committee at Hubei Women and Children’s Hospital. The research protocol for data analysis in this study was submitted to the Massachusetts General Hospital Institutional Review Board (IRB) on 18 August 2022. The protocol received approval under the IRB Number: 2022P002152.

### 2.3 EEG data acquisition

The EEG data acquisition process utilized BrainMaster Discovery 24 system, designed to capture EEG signals spanning from DC 0 Hz to 80 Hz, boasting 24-bit precision. Application of up to 24 scalp electrodes was meticulously performed using collodion and adhering to precise measurement protocols. The sampling frequency was 256 Hz. The participants were required to sit in a comfortable chair in a quiet room for about 5 min. EEG recordings were conducted during resting but wakeful state and no visual cues were presented to the participants. Participants were instructed to close their eyes during the recording; however, due to the young age of the participants, most were unable to follow the instructions and keep their eyes closed for the entire duration of the EEG measurement. The subsequent analytical focus was centered on 19 channels available for all participants with channel placement following the international 10–20 system. This set of channels encompassed *Fp1*, *Fp2*, *F7*, *F3*, *Fz*, *F4*, *F8*, *T3*, *C3*, *Cz*, *C4*, *T4*, *T5*, *P3*, *Pz, P4*, *T6*, *O1*, and *O2* with A1 being the reference channel at left mastoid.

### 2.4 Data preprocessing

The EEG preprocessing process was primarily carried out using the MNE-python package version 1.3.1 (Larson et al., 2022). First, a notch filter was employed to eliminate the 50 Hz line noise caused by powerline interference. Following this step, a bandpass filter ranging from 0.5 Hz to 42 Hz was applied. Next, average referencing was applied as the re-referencing method.

To address problematic channels, EEGLAB tool (Delorme and Makeig, 2004) was employed. Channels were designated as “bad” and consequently removed under specific criteria: channels with flat activity for over 5 s, those exhibiting a high-frequency noise standard deviation lower than 4.5 μV, and channels displaying a correlation lower than 0.7 with neighboring channels (SCCN, 2023). Subsequently, EEG waveforms were plotted in the time domain and subjected to visual inspection. As a result of the visual inspection, 6 participants characterized by extreme EEG artifacts were excluded from subsequent analyses.

The EEG waveforms were then divided into 10-s epochs, each with a 2-s overlap. Any epochs deemed “bad” were excluded from further analysis if their maximum peak-to-peak signal amplitude exceeded 10,000 μV or their minimum peak-to-peak signal amplitude fell below 0.1 μV. Finally, 4 participants with total length less than 1 min after removal of bad epochs were excluded from subsequent analyses. The final number of EEG samples that went into the analysis was 246 samples for ASD and 42 samples for control.

### 2.5 Feature extraction

Since our approach is a data-driven approach, we included a variety of EEG features. Five frequency bands were defined as delta (1–4 Hz), theta (4–8 Hz), alpha (8–13 Hz), beta (13–30 Hz), and gamma (30–42 Hz). EEG features were extracted from each epoch and averaged across all epochs and 1,046 features were extracted in total from the EEG of each participant. Spectral power for all five bands and each channel combination was first obtained using multi-taper spectral estimation (Slepian, 1978), where we used 7 tapers and therefore having a frequency resolution of 0.4 Hz. Subsequently, relative spectral power was calculated by normalizing the spectral power over the total power (0.5-42 Hz), yielding values between 0 and 1. A total of 95 relative spectral power features were computed.

Channel-to-channel spectral coherence was calculated for each of the 5 bands. 171 channel-to-channel spectral coherence features were computed for each band and a total of 855 spectral coherence features were computed for all 5 bands in total. The computation of spectral coherence features was done using the spectral_connectivity_epochs() function from the mne_connectivity package (MNE-Connectivity, 2023).

Several common statistical features in time-domain—sample entropy, skew, kurtosis, standard deviation, and mean—were all calculated for each of the 19 channels using respective functions from the mne-features package (Schiratti et al., 2018). This yielded a total of 95 common time-domain statistical features. Lastly, a binary feature was computed to check whether a participant’s EEG has alpha band using a method (Corcoran et al., 2018) and associated software (Corcoran et al., 2019) that quantifies individual alpha frequency.

### 2.6 Statistical inference

Here, the EEG feature served as the outcome variable, reflecting the dependent variable within the regression model. On the other hand, ASD/non-ASD represented the exposure variable, serving as the independent variable in the regression analysis. Additionally, demographic variables were integrated as covariates in the model to reduce the effects of confounding.

To further mitigate potential confounding effects stemming from the demographic variables, a propensity model based on logistic regression was executed. This facilitated an optimal full match (Hansen and Klopfer, 2006) procedure through the utilization of the MatchIt package (Ho et al., 2011) in R. The resultant matching weights obtained from the optimal full match were then incorporated as propensity score weights (Greifer, 2023) within the regression models using the Survey package (Lumley, 2023) in R. The entire process encompassed the execution of a total of 1,046 regression models, each corresponding to a distinct EEG feature that had been extracted. For a detailed explanation of the optimal full match and the utilization of matching weights in the regression models, please refer to Supplementary Data File 1.

**Figure 1:**
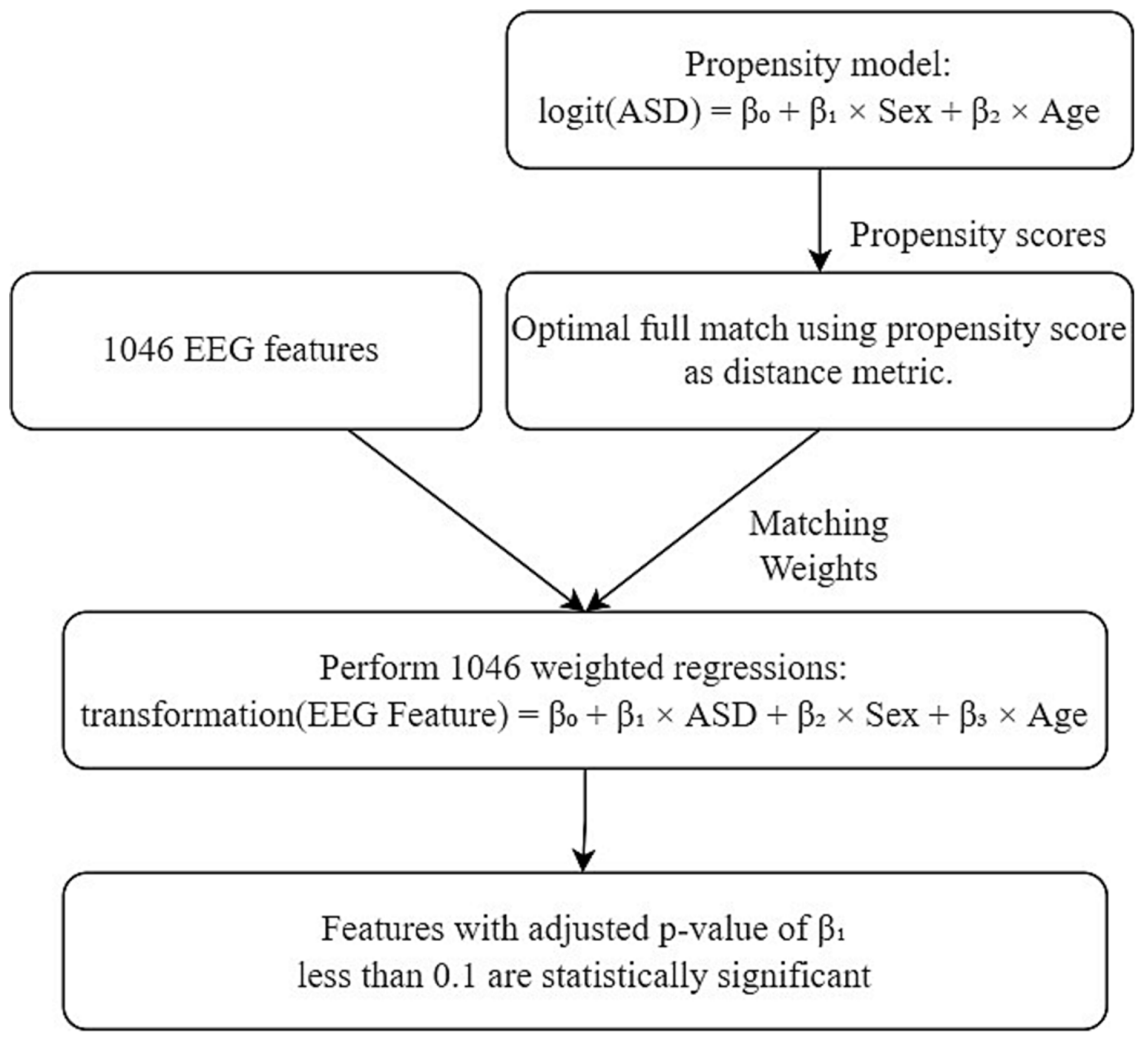
Statistical inference workflow. ASD represents the binary variable for ASD/control. Transformation applied to EEG feature is dependent on feature type.

Within this framework, logarithmic transformations were applied to EEG feature variables with strictly positive values, while EEG feature variables ranging between 0 and 1 underwent logit transformations. The coefficients linked to the exposure variable (ASD/non-ASD) were computed for every regression model, accompanied by their respective value of ps. Specifically, EEG features with Bonferroni-adjusted value of p for exposure variable coefficients lower than 0.1 were designated as statistically significant. Figure 1 depicts the entire inference workflow.

### 2.7 Machine learning classification

After the statistical inference, a leave-one-out-cross-validation (LOOCV) was performed where the entire inference step was nested within each split. That is during each split, the inference step is applied to each training set to select the top K features based on ranked Bonferroni adjusted *p*-values. Here we choose K = 15 based on a trade-off between (1) including as few features as possible to maintain a reasonable ratio between sample size and feature size to avoid overfitting; and (2) including as many features as possible to allow for the following nested forward feature selection.

The nested forward feature selection involved ridge logistic regression using the Scikit-learn package (Pedregosa et al., 2012). It was performed on each LOOCV training split using the top 15 features selected from the nested inference step. Notably, each ridge logistic regression was, in fact, a nested grid search to find the optimal alpha (i.e., regularization strength) that maximizes ROC-AUC. Moreover, to address the high-class imbalance, the minority class was upweighted in the loss function of the ridge logistic regression model. The features that achieved best nested cross validation performance were then used to fit another ridge logistic regression model, employing grid search on the training split to determine the optimal alpha. Subsequently, this model was used for inference on the single sample left out. We have included code to perform the aforementioned LOOCV procedure in Supplementary Data File 2.

### 2.8 Univariate classification of ASD using individual EEG features

In addition, each of the statistically significant features from the statistical inference step were used to classify ASD/non-ASD as standalone features for male and female participants separately, and an optimal threshold was chosen on the receiver operator characteristic curve (ROC) using the point closest to the top-left point of the ROC plot with perfect sensitivity and specificity.

## 3 Results

### 3.1 Comparison of EEG features in ASD vs. control

**Figure 2:**
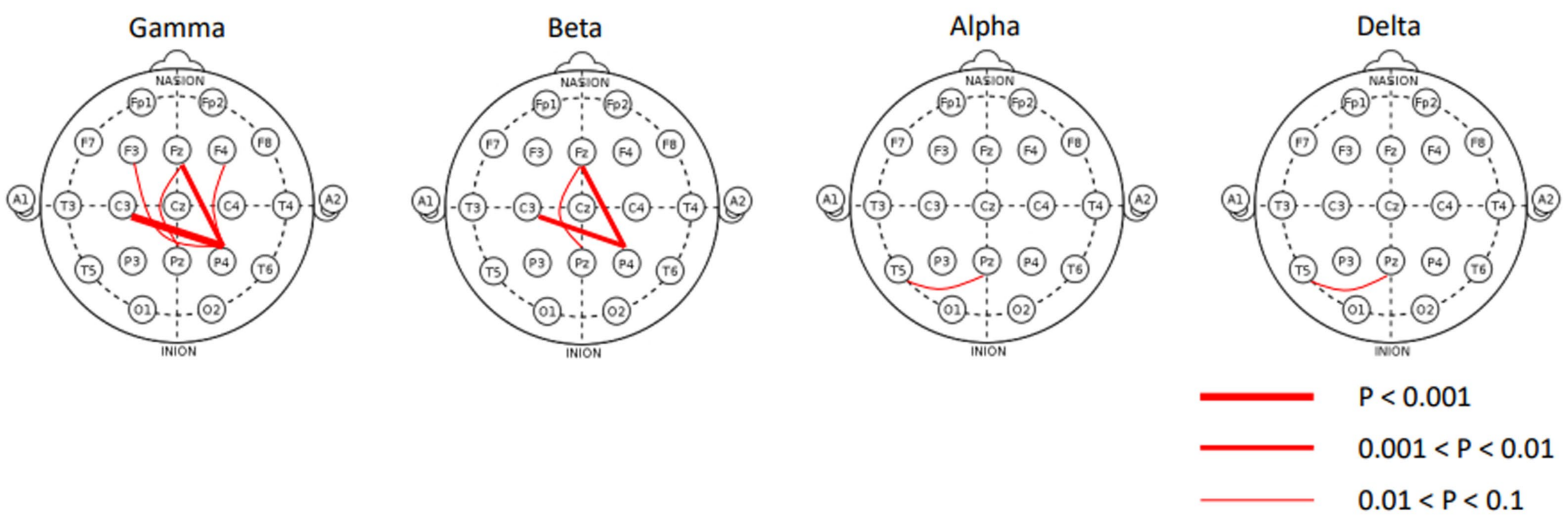
Statistically significant channel-to-channel spectral coherence features plotted on standard 10–20 montage. All connection lines indicate hyperconnectivity.

The covariates were balanced after optimal full matching (Supplementary Table 1), which reduced confounding bias in the covariates. Out of the 1,046 regression models analyzed in the matched cohort, only 10 exhibited a Bonferroni-adjusted value of p of less than 0.1 for the coefficient related to the binary ASD/non-ASD variable (Table 2). To facilitate a better understanding of the 0.1 significance level, we present histograms illustrating the distribution of raw *p*-values and their corresponding adjustments in Supplementary Figure 1. The 10 statistically significant EEG features exclusively consisted of spectral coherence features, all revealing elevated channel-to-channel spectral coherence within the ASD group. Notably, most instances of channel-to-channel spectral coherence were concentrated in the gamma and beta bands, as depicted in Figure 2. Within the gamma band, enhanced spectral coherence becomes evident among pairs like *P4* and *C3*, *P4* and *Fz*, *P4* and *F4*, *Pz* and *Fz*, as well as *Pz* and *Fz*. Similarly, in the beta band, augmented spectral coherence was observable between *P4* and *C3*, *P4* and *Fz*, and *Pz* and *Fz*. Moreover, the alpha and delta bands showed statistically significant spectral coherence between *Pz* and *T5*. No statistically significant spectral coherence was observed in the theta band.

**Table 2:** Statistically significant EEG features.

EEG feature	Coefficient	Std error	*N* (ASD)	*N* (control)	Adjusted *p*-value
Gamma band spectral coherence between P4 and C3	0.81	0.16	224	38	0.00047
Gamma band spectral coherence between P4 and Fz	0.81	0.18	215	36	0.0065
Beta band spectral coherence between P4 and C3	0.63	0.14	224	38	0.0078
Beta band spectral coherence between P4 and Fz	0.73	0.16	215	36	0.0098
Alpha band spectral coherence between Pz and T5	0.63	0.14	199	33	0.013
Gamma band spectral coherence between Pz and Fz	0.91	0.21	194	35	0.017
Delta band spectral coherence between Pz and T5	0.69	0.16	199	33	0.022
Beta band spectral coherence between Pz and Fz	0.75	0.18	194	35	0.036
Gamma band spectral coherence between P4 and F4	0.67	0.17	205	37	0.088
Gamma band spectral coherence between P4 and F3	0.68	0.17	209	37	0.091

### 3.2 Machine learning classification of ASD using EEG features

**Figure 3:**
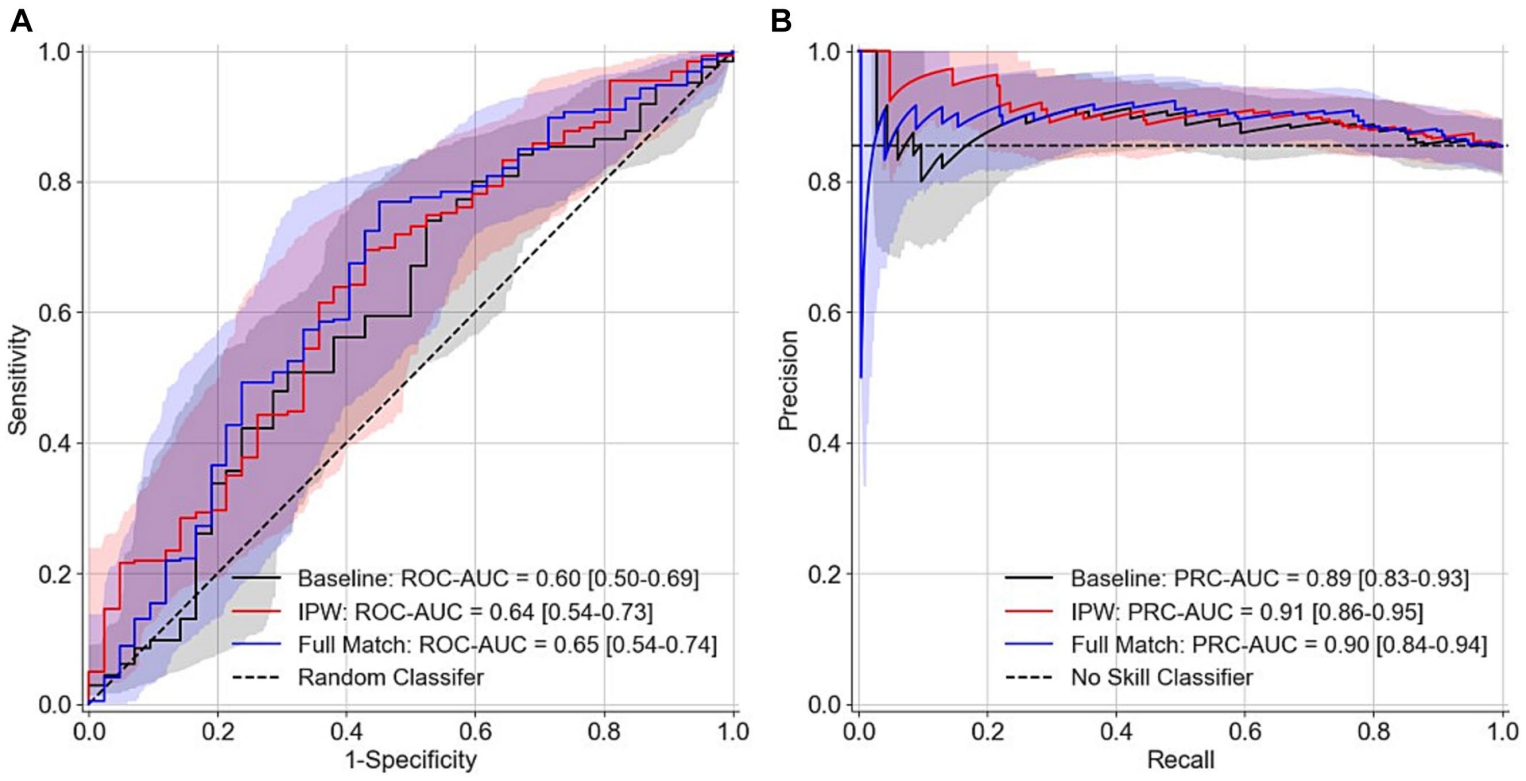
ROC curves and PRC curves resulting from machine learning LOOCV. **(A)** ROC curves **(B)** PRC curves. Baseline model represents a model that only uses age and sex as features. IPW and full match (i.e., optimal full match) represent different methods used in the nested inference step for feature selection. No skill model in panel **(B)** represents a model that classifies every participant as ASD. Bootstrapped confidence intervals for AUC curves are depicted by the shaded colors.

**Figure 4:**
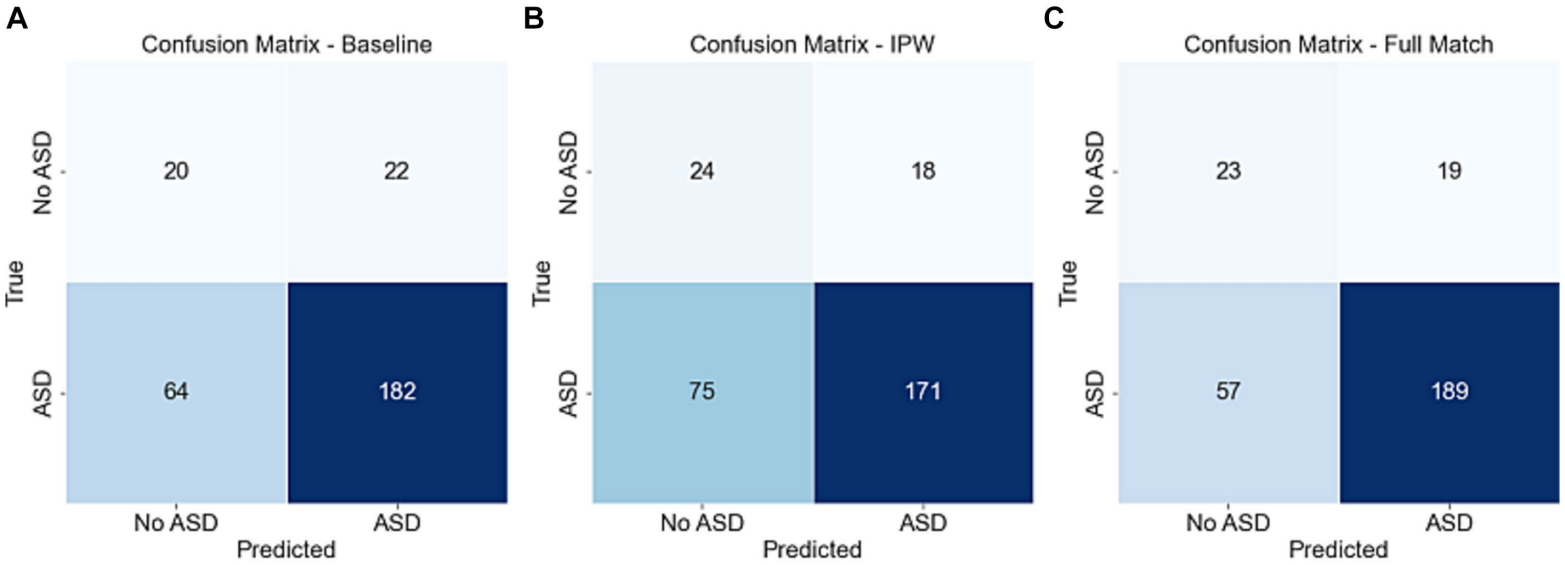
Confusion matrices resulting from machine learning LOOCV. **(A)** Confusion matrix for baseline model. **(B)** Confusion matrix for the model that uses IPW in the nested inference step for feature selection. **(C)** Confusion matrix for the model that uses full match in the nested inference step for feature selection.

The area under the receiver operating characteristic curve (ROC-AUC) from the LOOCV are 0.6 (95% confidence interval 0.50–0.69), 0.64 (0.54–0.73), and 0.65 (0.54–0.74) respectively for the baseline model that only included age and sex as features, the model that used inverse propensity score weighting (IPW) in the nested inference step for feature selection, and the model that used optimal full match in the nested inference step for feature selection (Figure 3A). The precision-recall curve AUC (PRC-AUC) from LOOCV for the three models are 0.89 (0.83–0.93), 0.91 (0.86–0.95), and 0.90 (0.84–0.94), respectively, for the three models (Figure 3B). The high PRC-AUCs are due to a 6 to 1 class imbalance of ASD participants (class 1) to control participants (class 0). Our models with EEG features only resulted in, at best, a 0.05 increase in ROC-AUC and a 0.02 increase in PRC-AUC. Moreover, the positive predictive value is calculated to be (0.89, 0.90, 0.91) and negative predictive value is calculated to be (0.24, 0.24, 0.29) for the baseline model, the model that used optimal full match in the nested inference step for feature selection, and the model that used IPW in the nested inference step for feature selection. Figure 4 depicts the confusion matrices from the LOOCV for the three models.3.3 Univariate Classification of ASD using Individual EEG Features.

Since machine learning classification using EEG features as predictive features only moderately enhanced model performance, we performed univariate analysis of the top 10 statistically significant EEG features to classify ASD/non-ASD for male and female participants separately. Supplementary Figure 2 depicts statistically significant spectral coherence feature plotted against CARS numeric total score for male and female separately, with the vertical line indicating the optimal cutoff. Although there is no statistically significant correlation between the 10 spectral coherence features with CARS numeric total score after Bonferroni correction, several spectral coherence features did exhibit relatively good discriminative performance for specific sex. For example, gamma band spectral coherence between *Pz* and *Fz* achieved sensitivity of 0.7 and specificity of 0.64 for male participants (Supplementary Figure 2J), and delta band spectral coherence between *Pz* and *T5* achieved sensitivity of 0.75 and specificity of 0.78 for female participants (Supplementary Figure 2E).

## 4 Discussion

We extracted 1,046 features from the EEG of 246 children with ASD and 42 control children in a relaxed eye-opened condition. Statistical inference revealed only increased functional connectivity which was concentrated mostly in the gamma and beta frequency bands, but also in delta and alpha bands. Machine learning classification using a LOOCV framework showed that the statistically significant EEG features only moderately enhanced classification performance (0.05 increase in AUC).

Among the 10 statistically significant channel-to-channel spectral coherence features we measured, five of them pertain to the gamma band. This aligns with multiple studies that have previously reported gamma band abnormalities in individuals with ASD (Sheikhani et al., 2012; Rojas and Wilson, 2014). We observed increased long-range gamma coherence between frontal and parietal lobe channels for the ASD participants in both the statistical inference approach and the univariate approach. Although most previous studies have primarily reported reduced long-range spectral coherence in ASD (Perez Velazquez et al., 2009; Khan et al., 2013), a few studies have also reported solely increased coherence both short range and long range (Sheikhani et al., 2012; Wang et al., 2020). Long-range connectivity is a higher-level brain function that allows different brain regions to quickly coordinate and integrate information, enabling coherent behavioral and cognitive responses (Wang et al., 2020). The increased coherence observed in ASD participants may indicate a failure of developmentally appropriate pruning or die-back and may interfere with normal cortical processing. Alternatively, the increased coherence may represent a compensatory attempt of the autistic brain which leads to the formation of atypical, spatially disparate, cortical networks in an attempt to replace function normally subserved by assumed-to-be deficient more localized networks (Duffy and Als, 2012). Moreover, weak central coherence (WCC) is a special feature and in-built cognitive style of ASD (Happé and Frith, 2006; Bojda et al., 2021) presenting difficulty to form meaningful links from similar parts such as generalizing forest from individual trees. In other words, ASD patients stay super focused on certain details such as an individual tree which reflects on the hyperconnectivity or increased coherence between certain brain regions to specific stimuli. This could be the roof of restricted interests and repetitive behaviors (RRB) which is one of the two core symptoms of ASD. RRB is critical for ASD diagnosis (Hyman et al., 2020) and its early restrictive interest in some non-social objects contributed to the ASD’s another core symptom social communication deficit (Sasson and Touchstone, 2014). Two previous studies indicated that increased α connectivity at 14 months was associated with later ASD diagnosis and RRB severity (Orekhova et al., 2014; Haartsen et al., 2019). A recent study displayed a strong association between EEG functional connectivity and RRBs and suggests its potential utilization as a biomarker to differentiate individuals with and without ASD (Sun et al., 2023). The natural links of certain obsessive attentions and their corresponding hyperconnectivity pathways in different brain regions provide reliable early diagnosis and subtyping guidance.

There are other studies that used machine learning to classify ASD vs. control group using resting state EEG recordings. One study used features derived from recurrence quantification analysis (Heunis et al., 2018) methods to classify ASD vs. controls and achieved high (0.97) accuracy in an approximately 1:1 ASD to control sample size (Bosl et al., 2017). Another study combined spectral power and eye tracking features for classification and achieved a good (0.93) AUC (Kang et al., 2020). However, a similar study also used power spectrum and functional connectivity features for classification also achieved poor results (accuracy 47%–57%; Garcés et al., 2022). These disparities in classification performances could be attributed to reasons such as differences EEG instruments, processing pipeline, feature extraction procedures, sample sizes, participant demographics, and machine learning methodologies etc. One methodology used in our study worth noting is combining optimal full matching and outcome regression analysis in the statistical inference. Matching is a non-parametric way of ensuring that the treated and control groups are similar in terms of subpopulation demographics which reduces confounding bias during group comparison of EEG features. After matching, the use of outcome regression increases precision in the effect estimates. Moreover, nesting the inference step inside LOOCV as the feature selection method facilitates a feature selection that is based on statistical significance without leaking information into the test splits.

In the future, we plan to use novel machine learning methods such as creating pre-trained deep learning foundation model on publicly available large EEG datasets and then fine-tune for ASD classification on this dataset.

### 4.1 Limitations

Several limitations exist in the study. First, the female sample is drastically smaller than the male sample size (44 vs. 244). There is also a big relative imbalance in sex where 87% of the participants with ASD are male and only 13% of ASD participants are female. Although propensity score matching was used to balance the subpopulation and demographic covariates were adjusted for in the outcome regression models, potential bias could still exist. Moreover, the choice of excluding eyes-closed participants significantly reduced our participant sample size for data analysis. In addition, the study follows a cross-sectional study design which has inherent weaknesses such as being prone to sampling bias and potential confounders (Wang and Cheng, 2020). Furthermore, the recruitment strategy resulted in a substantial disparity between ASD and control participants, with a disproportionate representation of ASD participants due to challenges in obtaining consent from parents of children in the control group. The reluctance of parents with healthy children to participate led to a significantly lower number of control participants, introducing a substantial 6 to 1 class imbalance. The class imbalance could have significant impact on both the machine learning model performance and the regression coefficient estimates during statistical inference, due to the potential of the models biasing the majority class (Luque et al., 2019).

Another major limitation of the paper is not having a held-out test set (external validation) that was never used in any model training, tuning, and feature selection. We used nested LOOCV during machine learning classification of ASD due to the small sample size; however, it poses risks for potential leakage, overfitting, and inflated estimation of model performance. The risk of overfitting and inflated performance from cross validation has been reported in multiple systematic reviews of machine learning classification in neurological and developmental disorders (Pulini et al., 2019; Vabalas et al., 2019). Even though we nested feature selection in the training splits of LOOCV, our approach could still be considered a form of circular analysis or “peeking” (Pulini et al., 2019). This is because statistical analysis was performed on the entire dataset prior to performing machine learning classification and statistical analysis helped to inform the rough number (i.e., threshold) of features to pass to the nested forward selection step during LOOCV. Thus, the reported machine learning classification performance in this paper could be inflated.

Lastly, CARS does not have sub-scores of social deficits and RRB as Autism Diagnostic Observation Schedule (ADOS) does, so that we were unable to make direct correlation between our increased coherence and RRB which we believe they could be very likely correlated.

## Data availability statement

The dataset used in this research is protected information that is not publicly available. Requests to access the datasets should be directed to xkong1@mgh.harvard.edu.

## Ethics statement

The studies involving humans were approved by Ethics Committee at Hubei Women and Children’s Hospital and Massachusetts General Hospital Institutional Review Board. The studies were conducted in accordance with the local legislation and institutional requirements. Written informed consent for participation in this study was provided by the participants’ legal guardians/next of kin. Written informed consent was obtained from the individual(s), and minor(s)’ legal guardian/next of kin, for the publication of any potentially identifiable images or data included in this article.

## Author contributions

SK: Formal analysis, Investigation, Methodology, Software, Validation, Visualization, Writing – original draft. HW: Data curation, Writing – review & editing. HS: Investigation, Methodology, Software, Writing – review & editing. AZ: Data curation, Writing – review & editing. JL: Data curation, Writing – review & editing. XZ: Data curation, Writing – review & editing. KL: Project administration, Writing – review & editing. MW: Methodology, Supervision, Writing – review & editing. HX: Funding acquisition, Supervision, Writing – review & editing. X-jK: Funding acquisition, Project administration, Supervision, Writing – review & editing.
