# Corrigendum: Classification of autism spectrum disorder using electroencephalography in Chinese children: a cross- sectional retrospective study

**DOI:** 10.3389/fnins.2024.1501782

**Published:** 2024-10-29

**Authors:** Si Yang Ke, Huiwen Wu, Haoqi Sun, Aiqin Zhou, Jianhua Liu, Xiaoyun Zheng, Kevin Liu, M. Brandon Westover, Haiqing Xu, Xue-jun Kong

**Affiliations:** ^1^Anthinoula A. Martinos Center, Massachusetts General Hospital, Charlestown, MA, United States; ^2^Hubei Maternity and Child Health Hospital, Wuhan, Hubei, China; ^3^Department of Neurology, Beth Israel Deaconess Medical Center, Boston, MA, United States; ^4^Huangshi Maternity and Child Health Care Hospital, Huangshi, Hubei, China; ^5^Department of Biomedical Informatics, Harvard Medical School, Boston, MA, United States; ^6^Department of Neurology, Massachusetts General Hospital, Boston, MA, United States; ^7^Department of Psychiatry, Beth Israel Deaconess Medical Center, Boston, MA, United States

**Keywords:** autism spectrum disorder, electroencephalography, machine learning, spectral power, functional connectivity, coherence

In the published article Dr. Westover's disclosure statement was inadvertently omitted. The disclosure should read:

