# Supplementary material for: Classification of autism spectrum disorder using electroencephalography in Chinese children: a cross-sectional retrospective study
Source: Front Neurosci. 2024 Jan 25;18:1330556. doi: 10.3389/fnins.2024.1330556 (PMC10850305; doi:10.3389/fnins.2024.1330556)
Supplement: Supplementary file 1 [file Data_Sheet_1.DOCX]

Supplementary Material

**Classification of Autism Spectrum Disorder using Electroencephalography in Chinese Children: A Cross-sectional Retrospective Study**

**Si Yang Ke^1,3†^, Huiwen Wu^2†^, Haoqi Sun^1,4^, Aiqin Zhou^2^, Jianhua Liu^6^, Xiaoyun Zheng^2^, Kevin Liu^1,3^, M. Brandon Westover^4,5*^, Haiqing Xu^2*^ , and Xue-jun Kong^1,7*^**

*** Correspondence:** xkong1@mgh.harvard.edu

**Explanation for optimal full match, stratum propensity scores, matching weights, and how they are used for inference in our outcome regression models.**

**1. Optimal Full Match**

Optimal full matching, as implemented in the function matchit() from the MatchIt R Package (Ho et al., 2011) with the method set to "full," involves a form of subclassification where all units, both treatment, and control, are assigned to a subclass and receive at least one match. The matching is optimal in the sense that the sum of the absolute distances between treated and control units in each subclass is minimized. This method is considered a robust alternative to propensity score weighting, as the propensity score model does not need to be correct to estimate the treatment effect without bias (*Optimal Full Matching — Method_full*, n.d.). For the detailed algorithm of the matching process, please see *Optimal Full Matching and Related Designs via Network Flows* (Hansen and Klopefer 2006).

**2. Stratum propensity scores**

Once each sample is assigned a stratum via the aforementioned optimal full match process, we can compute stratum propensity scores as the proportion of treated units in that stratum such that stratum propensity score = $\frac{n1s_{i}}{ns_{i}}$ where $n1s_{1}$ is the number of treat units in stratum $s_{i}$ and $ns_{i}$is the total number of units in stratum $s_{i}$ (Greifer 2023).

**3. Matching weights**

From the stratum propensity scores, we can just apply straightforward propensity score formulas to get the matching weights. Because we are interested in the average treatment effect, the formulas are as follows (Greifer 2023):

- Treated (i.e. ASD) subjects receive a weight of $\frac{ns_{i}}{n1s_{i}}$
- Control subjects receive a weight of $\frac{ns_{i}}{n0s_{i}}$

**4. Code snippet to perform optimal full match and using the matching weights as weights in a single outcome regression model using the feature delta band power at Fp1 channel as an example:**

***creating propensity score model and performing optimal full match***

*library(matchit)*

*fullmatch.out <- matchit(*

*trt ~ age_months + sex, # trt = ASD/control*

*data = features_df, # 288 (subject count) by 1046 (feature count) features matrix*

*method = "full", # optimal full match*

*distance = 'glm',*

*estimand = "ATE"*

*)*

*matched.data <- match.data(fullmatch.out)*

***Helper function to perform a single outcome regression model in the form of***

***phenotype (feature) ~ exposure (ASD/control) + covariates***

library(survey)

*ipw_svyglm <- function(phenotype="bp_delta_Fp1", exposure="trt", covariates=c("sex", "age_months"), data=matched.data, transformation = 'logit') {*

*mod_design <- svydesign(id =~ 1, weights = ~ weights, data = data)*

*# create outcome model: phenotype ~ trt + sex + age_months*

*covariate_string <- paste(covariates, collapse="+")*

*if (transformation == 'logit') {*

*mod_string <- sprintf('log(%s/(1-%s)) ~ %s + %s', phenotype, phenotype, exposure, covariate_string)*

*mod <- svyglm(as.formula(mod_string), design = mod_design, family = gaussian())*

*}*

*return (mod)*

*}*

**References**

1. Ho, D. E., Imai, K., King, G., & Stuart, E. A. (2011). MatchIt: Nonparametric Preprocessing for Parametric Causal Inference. *Journal of Statistical Software, 42*(8). doi:10.18637/jss.v042.i08

2. *Optimal Full Matching—Method_full*. (n.d.). Retrieved November 29, 2023, from https://kosukeimai.github.io/MatchIt/reference/method_full.html#references-1

3. Ben B Hansen & Stephanie Olsen Klopfer (2006) Optimal Full Matching and Related Designs via Network Flows, Journal of Computational and Graphical Statistics, 15:3, 609-627, DOI: [10.1198/106186006X137047](https://doi.org/10.1198/106186006X137047)

4. Matching Weights are Propensity Score Weights | Noah Greifer. (2023). Retrieved September 6, 2023, from <https://ngreifer.github.io/blog/matching-weights/>
