# Supplementary material for: Classification of autism spectrum disorder using electroencephalography in Chinese children: a cross-sectional retrospective study
Source: Front Neurosci. 2024 Jan 25;18:1330556. doi: 10.3389/fnins.2024.1330556 (PMC10850305; doi:10.3389/fnins.2024.1330556)
Supplement: Supplementary file 2 [file Data_Sheet_2.DOCX]

Supplementary Material

**Classification of Autism Spectrum Disorder using Electroencephalography in Chinese Children: A Cross-sectional Retrospective Study**

**Si Yang Ke^1,3†^, Huiwen Wu^2†^, Haoqi Sun^1,4^, Aiqin Zhou^2^, Jianhua Liu^6^, Xiaoyun Zheng^2^, Kevin Liu^1,3^, M. Brandon Westover^4,5*^, Haiqing Xu^2*^ , and Xue-jun Kong^1,7*^**

*** Correspondence:** xkong1@mgh.harvard.edu

**Python code for performing the LOOCV for machine learning classification.**

**1. Helper function logistic_regression_netsed_cv() that is used in forward selection.** It performs a 10 × 10 nested cross validation (CV) using logistic regression where the where the inner CV selects the best alpha (regularization strength) based on ROC-AUC highest. The mean ROC AUC of the outer CV is returned.

from sklearn.model_selection import GridSearchCV, RepeatedStratifiedKFold

from sklearn.impute import SimpleImputer, KNNImputer

from sklearn.metrics import recall_score, precision_score, roc_auc_score

from sklearn.linear_model import LogisticRegression

import numpy as np

def logistic_regression_nested_cv(feature_list, X_train,y_train):

“”“ This function is used by forward selection, it takes in a

feature subset and performs 10 x 10 nested CV using logistic regression.

The mean CV ROC-AUC is returned”””

    X_train = X_train[feature_list]

    numeric_cols = [col for col in feature_list if col not in [ 'sex']]

    categorical_cols = ['sex']

# Initialize the array to store the nested scores

nested_roc_auc = np.zeros(10)

    # Define the indices for the outer loop splits

    outer_cv = StratifiedKFold(n_splits=10, random_state=7, shuffle=True)

    outer_cv_splits = outer_cv.split(X_train, y_train)

    # Loop over the outer loop splits

    for j, (train_val_idx, train_test_idx) in enumerate(outer_cv_splits):

        # Split the data into training/validation and testing sets

        X_train_val, y_train_val = X_train_val.loc[train_val_idx,:], y_val[train_val_idx]

        X_train_test, y_train_test = X_train_val.loc[train_test_idx,:], y_val[train_test_idx]

        steps = [('impute', SimpleImputer(strategy='mean'))]

        pipeline = Pipeline(steps=steps)

        # Apply the pipeline on the training/validation set

        X_train_val = pipeline.fit_transform(X_train_val)

        # Apply the pipeline on the testing set

        X_test_val = pipeline.transform(X_test_val)

        # Define the indices for the inner loop splits

        inner_cv = RepeatedStratifiedKFold(n_splits=10, random_state=42, n_repeats=1)

        # Define the estimator and the parameter grid for GridSearchCV

        lr = LogisticRegression(class_weight='balanced',max_iter=1000)

        param_grid = {'C': [0.0001,0.001, 0.01, 0.1, 1, 10, 100]}

        clf = GridSearchCV(estimator=lr, param_grid=param_grid, cv=inner_cv,n_jobs=-1,scoring='roc_auc')

        # Fit the estimator on the training/validation set and calibrate the predictions

        clf.fit(X_train_val, y_train_val)

        # find the best cutoff

        roc_auc = roc_auc_score(y_train_test, clf.predict_proba(X_train_test)[:, 1])

        # Store the scores in the array

        nested_roc_auc[j] = roc_auc

    return nested_roc_auc.mean()

**2. Function forward_selection() that performs forward selection given the top 15 features from the nested inference step. It iteratively adds features from the top 15 feature list to the current set of features and evaluates the performance using logistic regression with nested cross-validation. The best set of features is then determined based on the maximum mean ROC AUC.**

def forward_selection(X_train, y_train,top_15_features):

    all_mean_roc_auc = []

    for i in tqdm(range(len(top_15_features))):

        features = top_15_features[:i+1]

        features = features + ['age_months','sex']

        all_mean_roc_auc.append(logistic_regression_nested_cv(features,X_train,y_train))

    best_index = np.argmax(all_mean_roc_auc)

    best_features = top_15_features[:best_index+1] + ['age_months', 'sex']

    return best_features, all_mean_roc_auc

**3. Main for loop for performing LOOCV that uses the two functions defined previously in 1 and 2.**

from sklearn.model_selection import LeaveOneOut

from sklearn.preprocessing import LabelEncoder

y_true_list = []

y_pred_proba_list = []

y_pred_list = []

y = df['ASD']

# store features as X

X = df.copy(deep=True)[features_names]

# label encode the sex column

le = LabelEncoder()

X['sex'] = le.fit_transform(X['sex'])

loo = LeaveOneOut()

# Loop over the outer loop splits

for j, (train_idx, test_idx) in enumerate(loo.split(df)):

    # Split the data into training/validation and testing sets

    train = df.loc[train_idx,:]

    X_train, y_train = X.loc[train_idx,:], y[train_idx]

    X_test, y_test = X.loc[test_idx,:], y[test_idx]

    features = X_train.columns

    # save X_train_val to a csv file

    train_val.to_csv('train.csv',index=False)

    # Run the IPW or Optimal Full Match to get list of top 15 features

    print("Generating Ranked P-value List From R")

    args = ["Rscript", "get_significance_list_optimized.R", "full_match"]

    res = subprocess.run(args, stdout=subprocess.PIPE, stderr=subprocess.PIPE)

    try:

        significance_ranking = pd.read_csv('ranking_list.csv')

        top_15_features = significance_ranking.outcome.tolist()

    except FileNotFoundError:

        assert False, "File not found!"

# Forward selection

    best_features, all_mean_roc_auc = forward_selection(X_train_val.reset_index(drop=True),y_train_val.reset_index(drop=True),top_15_features)

    X_train = X_train[best_features]

    X_test = X_test[best_features]

    steps = [('mean_impute', SimpleImputer(strategy='mean'))]

    pipeline = Pipeline(steps=steps)

    # Apply the pipeline on the trainin

    X_train = pipeline.fit_transform(X_train)

    # Apply the pipeline on the testing set

    X_test = pipeline.transform(X_test)

    # define inner cv

    inner_cv =  RepeatedStratifiedKFold(n_splits=10, random_state=42, n_repeats=3)

    # Define the estimator and the parameter grid for GridSearchCV

    lr = LogisticRegression(class_weight='balanced',max_iter=1000)

    param_grid = {'C': [0.0001,0.001, 0.01, 0.1, 1, 10, 100]}

    clf = GridSearchCV(estimator=lr, param_grid=param_grid, cv=inner_cv,n_jobs=-1,scoring='roc_auc')

    clf.fit(X_train, y_train)

    nested_C = clf.best_params_['C']

    y_pred = clf.predict(X_test)

    y_pred_proba = clf.predict_proba(X_test)[:,1]

    y_true_list.extend(y_test)

    y_pred_proba_list.extend(y_pred_proba)

    y_pred_list.extend(y_pred)

    old_significance_ranking = significance_ranking

    print("Finished LOOCV Iteration ",j)
