# Supplementary material for: Classification of autism spectrum disorder using electroencephalography in Chinese children: a cross-sectional retrospective study
Source: Front Neurosci. 2024 Jan 25;18:1330556. doi: 10.3389/fnins.2024.1330556 (PMC10850305; doi:10.3389/fnins.2024.1330556)
Supplement: Supplementary file 3 [file Table_1.DOCX]

**Supplemental Table 1.** Summary of balance of data after optimal-full match (Std. Mean Difference = (Mean(AD)-Mean(Non-ASD))/Pooled Standard Deviation, i.e., Cohen’s d).

|  | Mean (ASD) | Mean  (Non-ASD) | Std. Mean  Difference | Variance  Ratio for ASD/Non-ASD |
| --- | --- | --- | --- | --- |
| Age in Months | 36.90 | 36.48 | 0.03 | 1.46 |
| Sex Female | 0.15 | 0.16 | -0.01 |  |
| Sex Male | 0.85 | 0.84 | 0.01 |  |
| Distance  (Propensity Score) | 0.85 | 0.85 | -0.01 | 1.02 |
