# Supplementary figures and images for: Classification of autism spectrum disorder using electroencephalography in Chinese children: a cross-sectional retrospective study
Source: Front Neurosci. 2024 Jan 25;18:1330556. doi: 10.3389/fnins.2024.1330556 (PMC10850305; doi:10.3389/fnins.2024.1330556)

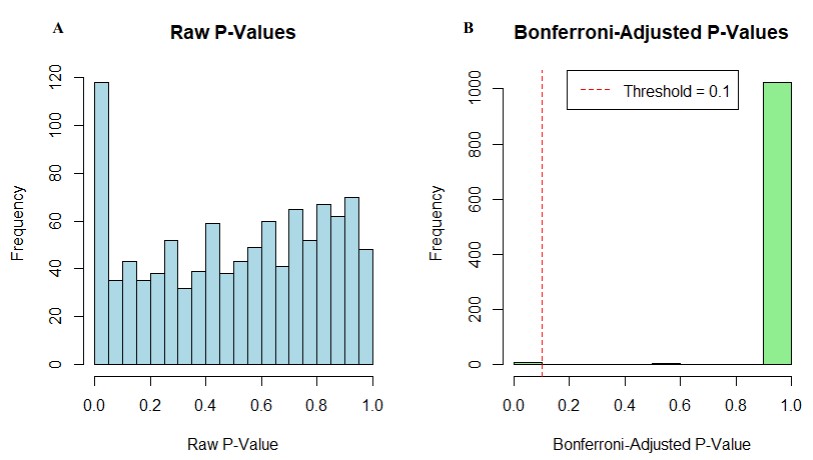

Supplement: Supplementary file 4 [file Image_1.JPEG]

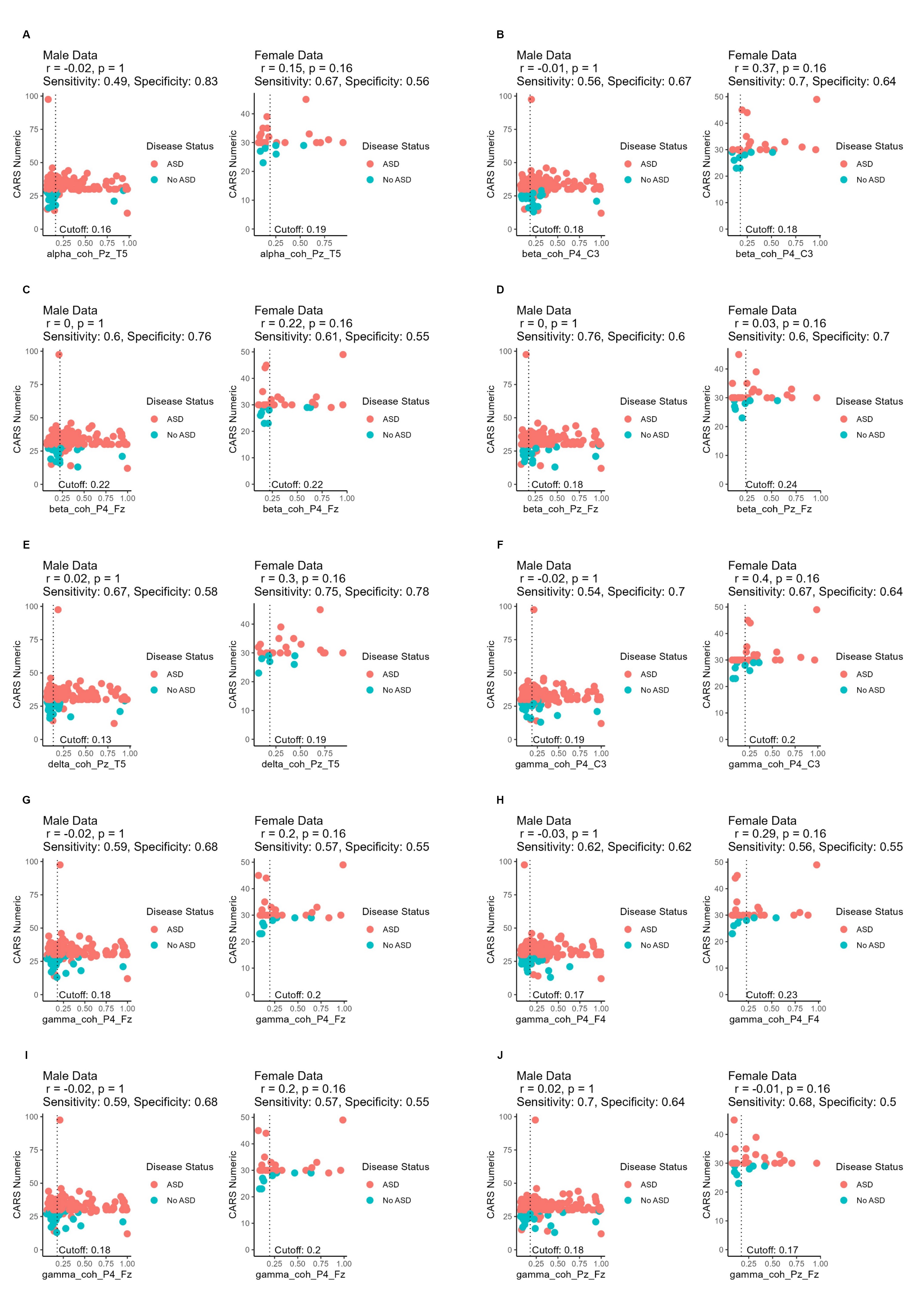

Supplement: Supplementary file 5 [file Image_2.JPEG]
